# Physiological Entrainment: A Key Mind–Body Mechanism for Cognitive, Motor and Affective Functioning, and Well-Being

**DOI:** 10.3390/brainsci15010003

**Published:** 2024-12-24

**Authors:** Marco Barbaresi, Davide Nardo, Sabrina Fagioli

**Affiliations:** Department of Education, “Roma Tre” University, 00185 Rome, Italy; marco.barbaresi@uniroma3.it (M.B.); sabrina.fagioli@uniroma3.it (S.F.)

**Keywords:** neural entrainment, rhythmic entrainment, EEG, embodied cognition, mind–body connection

## Abstract

Background: The human sensorimotor system can naturally synchronize with environmental rhythms, such as light pulses or sound beats. Several studies showed that different styles and tempos of music, or other rhythmic stimuli, have an impact on physiological rhythms, including electrocortical brain activity, heart rate, and motor coordination. Such synchronization, also known as the “entrainment effect”, has been identified as a crucial mechanism impacting cognitive, motor, and affective functioning. Objectives: This review examines theoretical and empirical contributions to the literature on entrainment, with a particular focus on the physiological mechanisms underlying this phenomenon and its role in cognitive, motor, and affective functions. We also address the inconsistent terminology used in the literature and evaluate the range of measurement approaches used to assess entrainment phenomena. Finally, we propose a definition of “physiological entrainment” that emphasizes its role as a fundamental mechanism that encompasses rhythmic interactions between the body and its environment, to support information processing across bodily systems and to sustain adaptive motor responses. Methods: We reviewed the recent literature through the lens of the “embodied cognition” framework, offering a unified perspective on the phenomenon of physiological entrainment. Results: Evidence from the current literature suggests that physiological entrainment produces measurable effects, especially on neural oscillations, heart rate variability, and motor synchronization. Eventually, such physiological changes can impact cognitive processing, affective functioning, and motor coordination. Conclusions: Physiological entrainment emerges as a fundamental mechanism underlying the mind–body connection. Entrainment-based interventions may be used to promote well-being by enhancing cognitive, motor, and affective functions, suggesting potential rehabilitative approaches to enhancing mental health.

## 1. Introduction

Rhythm is a universal phenomenon that can be observed in various natural environments [1], manifesting in physical (e.g., the oscillation of a pendulum clock), environmental (e.g., the pulsing of waves in the ocean), and biological systems (e.g., the migratory patterns of birds). In humans, rhythms can be observed in the rhythmicity of neural activity, which can be associated with the self-sustaining autonomic processes of the body, tablesuch as the heartbeat and breathing [2,3], sleep–wake cycles (a direct effect of circadian rhythms) [4], complex motor activity [5,6], and language processing [7].

Rhythm can also influence sensory processing [8]. For instance, visual and auditory perception in both animals and humans consists of a discrete sequence of perceptual cycles, rather than a continuous information processing stream [9]. Similarly, the central integration of sensory input is sensitive to cycling at precise brain frequencies, supporting the hypothesis of underlying rhythmic activity in sensory processing [10].

Research shows that both motor and sensory rhythms share common brain networks for temporal adaptation to enhance coordination skills [11]. For example, temporal brain regions are associated with sound processing and auditory speech perception [12], suggesting that selective attention can sample stimuli rhythmically [13], as seen in sustained attentional processing [14]. Therefore, specific neural rhythmic phenomena, such as those captured via electroencephalographic (EEG) frequency bands, can underlie different cognitive functioning [15]. Notably, some rhythmic brain oscillations are common across multiple species, despite variations in brain size, suggesting a fundamental role of rhythmic activity in guiding adaptive actions, decision-making, and social interactions [16]. Moreover, evidence shows that evolutionary changes in brain size do not necessarily alter the timing of brain rhythms, which are crucial for various cognitive functions [16].

Such considerations highlight the importance of investigating rhythmic activity across biological, environmental, and cognitive systems. However, the psychophysiological basis of the relationship between brain rhythmicity and human functioning remains a matter of debate, possibly due to the presence of inconsistencies in the definition of rhythmic physiological activity, as well as in the variability of measurement methods in the literature. Given the fundamental role of rhythmicity in human life [4], advancing our understanding of the psychophysiological correlates of human synchronization may contribute to understanding cognitive, motor, and affective functioning, and support the development of rhythm-based interventions aimed at enhancing well-being.

In this review, we explore the current literature on entrainment as a rhythmic psychophysiological phenomenon, focusing on its role in cognitive, motor, and affective functioning and considering only those studies that showed evidence of entrainment or synchronization phenomena. Specifically, we address entrainment in the contexts of auditory–motor integration, sensorimotor functioning and motor impairments, speech processing, and language disorders, as well as affective mechanisms involving empathy and social bonding. We also examine how different definitions and measurement approaches have influenced our understanding of physiological rhythmic processes, and discuss the theoretical models developed to interpret such phenomena. By considering the findings from different studies, we aim to clarify the role of entrainment under a new, unifying theoretical perspective, highlighting the potential applications of rhythm- and music-based interventions to support mental health and well-being.

## 2. Terminological Challenges in Entrainment Research

In rhythm research, *physiological synchronization* examines how individuals in interaction—whether working together or simply talking to each other—co-regulate their physiological responses through both central and autonomic nervous system activity [17,18,19,20]. Such a phenomenon reflects fundamental neural network activity and involves the bidirectional coupling of oscillations—a two-way interaction between oscillators, which can be defined as dynamic systems within biological or physical domains that produce rhythmic or cyclical activity over time [21]. For instance, it is a well-established fact that listening to speech modulates activity in the human motor cortex. This process, mostly observed when a person speaks, shows that external rhythmic stimuli can directly influence the neural processes involved in motor function [22]. In this case, the temporal patterns of speech emerge as a consequence of intrinsic rhythms in cortical areas [23]. Therefore, while activity in the motor cortex enables speech processing, the auditory cortex is thought to provide feedback about the sound that is being produced [24,25]. Such feedback can, in turn, adjust the motor output, creating an interaction in which both systems influence each other’s activity [21,26]. Conversely, unidirectional coupling occurs when one oscillator influences another in a one-way direction. In this scenario, the “driving oscillator” (i.e., the one providing the rhythmic input) affects the “receiving oscillator” (i.e., the one responding to the input), but not vice-versa. For example, when listening to a rhythmic sound, the auditory cortex may synchronize its oscillations with the rhythm of the sound but, obviously, the sound does not change as a consequence of brain activity. This effect can be observed when individuals are exposed to external rhythmic stimuli, such as music or a metronome’s beat. In this case, only the auditory cortex activity tends to synchronize its neural oscillations to the sound of the beat, and not vice-versa [27]. On these bases, the concept of *entrainment* is introduced as a specific form of one-way synchronization in which the temporal pattern of one rhythmic system influences and stabilizes another rhythmic system [28]. This process often involves a dominant oscillator—such as a pacemaker or external stimulus—which brings the rhythm of the secondary system into alignment with its cycle [27].

In contrast, general synchronization, which refers to events occurring simultaneously or coinciding in time, does not necessarily rely on oscillators as underlying rhythmic generators. This form of synchronization can also arise in chaotic systems and noisy environments, where coordinated timing emerges without a specific driving oscillator [29].

**Figure 1:**
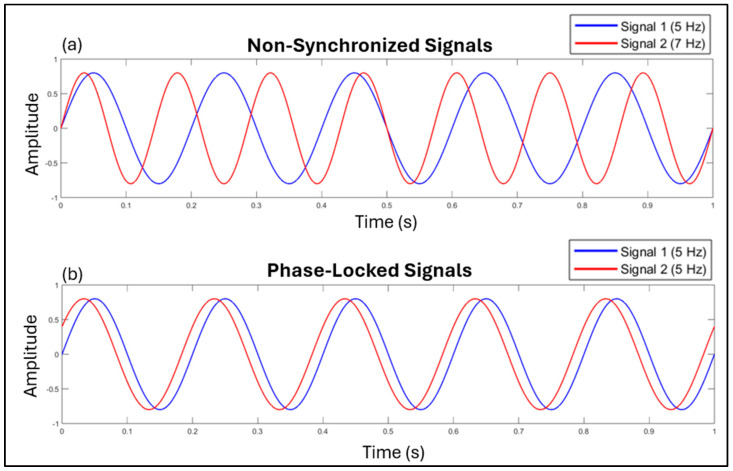
**Physics of Entrainment.** The upper panel (**a**), “Non-Synchronized Signals”, shows two oscillatory signals that are not synchronized. The blue signal has a frequency of 5 Hz, while the red signal has a frequency of 7 Hz. In the context of neural entrainment, non-synchronized signals indicate neural activities that are neither phase- nor frequency-aligned. These oscillations occur independently, with peaks and troughs appearing at different times, demonstrating a lack of coordination between the underlying neural pools. Such asynchrony suggests that the neural pools are either not entrained to the same external stimulus, or are not engaged in the same neural processing, resulting in uncoordinated oscillatory activity. The lower panel (**b**) “Phase-locked Signals” shows two phase-locked signals. The phase difference between two oscillators (or spike trains) remains constant over time (at 5 Hz for both signals). In this case, the individual spikes or oscillations do not necessarily occur at the same time, but the relationship between their phases is stable. For example, if two neurons fire spikes at different times but maintain a consistent phase difference, they are said to be *phase-locked* [31] or *coherently locked* (i.e., coherent state signals). According to entrainment models, phase-locked signals enable efficient information processing and propagation within brain networks, which can eventually extend into different bodily systems.

Therefore, the entrainment process emphasizes the importance of phase relationships and their biological adaptive significance, whereas synchronization, although based on the same phase-locking properties between signals, does not necessarily require an oscillator, and lacks the adaptive significance of entrainment. To further expand this concept, it is possible to claim that two events can be synchronized purely by coincidence—such as a person ducking at the same moment that someone shoots—without any underlying rhythmic coordination [30]. This argument suggests that, although entrainment and synchronization are functionally distinct processes, in physical terms they are closely related phenomena, as both can be represented by phase alignment between signals (see Figure 1).

Based on these considerations, it has been suggested that physiological synchronization may not fully capture the complexity of biological processes, although it refers to events occurring at the same phase angle [30]. This highlights the importance of considering entrainment as a key phenomenon in cognitive processes, affective functioning, and motor coordination [27].

For this reason, in this review, we adopted a psychophysiological perspective to examine entrainment across mind–body functions in the theoretical framework of embodied cognition, which posits that cognitive processes are shaped by reciprocal interaction between the body and its environment [32]. In this context, we adopt the term *physiological entrainment* to refer to a key supramodal mind–body mechanism that dynamically coordinates different bodily systems, supporting cognitive processing, motor coordination, and emotional functioning [21,33,34]. At the physical level, the entrainment phenomenon refers to the process of synchronizing our internal rhythmic processes to external regular stimuli or periodic cues (often associated with “beats”). Internal rhythmic processes are characterized as oscillators with particular temporal frequencies called periods. Stimulus onsets occurring at expected times are “in phase” with the internal oscillator. Conversely, onsets that occur before or after the expected time are oscillations that are “out of phase” [35].

The most prevalent form of physiological entrainment, observed at the neural level, is known as neural entrainment, and it is widely recognized as a key process supporting various physiological functions. For instance, neural entrainment is associated with consciousness and perception [36]; it is involved in regulating the activity of the autonomic nervous system [21] and in enhancing communication by supporting processes such as achieving meaning attributions for sensory information inflow [37].

Recent research has also investigated entrainment with regard to *physiological coherence* [38]. *Coherence* is defined as a higher state of synchronization across various oscillatory systems, including the heart–brain connection [39]. Such research suggests that achieving a state of higher physiological coherence in different bodily systems, such as heart-rate variability (HRV), sustains a widespread sine-wave-like rhythm activity, improving emotional self-regulation and cognitive performance. According to this model, physiological coherence is viewed as a widespread and higher-order state of entrainment that is characterized by stable and well-organized rhythmicity, particularly HRV rhythmic activity [38]. Notably, the idea that neurons can communicate, not only via firing rates but also via “communication through coherence”, was originally proposed by Fries [40]. According to that research, neural populations must fire simultaneously and in perfect alignment (i.e., in-phase synchronization) to achieve coordinated activity. We further discuss this theory in the next sections.

The concept of physiological entrainment involves a broader range of oscillators extending into the motor domain, whereby this state of synchronization manifests in actions such as coordinated movements or rhythmic social interactions [33], a manifestation that is mostly referred to as “social motor entrainment” [41]. In this context, it is also common to refer to *rhythmic entrainment* as a broader concept involving the synchronization of rhythmic activity in body systems with external oscillators. This process encompasses the entrainment of physiological systems with external stimuli such as music or rhythmic cues [42].

## 3. Quantifying Entrainment: Methods and Challenges

So far, we have outlined the distinction between synchronization and entrainment, focusing on their adaptive physiological relevance. Hence, entrainment involves the dynamic adjustment of internal oscillators to external environmental cues, also enabling optimal timing and adaptive function at the biological level [43]—as observed in cardiac cells, which synchronize their oscillations to generate a unified electrical signal that drives heart contractions [44,45].

However, in this section, we aim to explore how the entrainment phenomenon can be induced, investigated, and measured across different physiological systems, including neural brain activity, heart rate variability, and motor coordination and synchronization processes. 

Brain rhythmic oscillations can be easily triggered through various non-invasive rhythmic stimuli. For example, sensorimotor stimulation has been shown to synchronize brain oscillations with external rhythms [46,47] through rhythmic musical stimuli [48] and more generally rhythmic sensory stimulation [27,49]. Auditory stimulation, such as rhythmic sound patterns, similarly triggers neural entrainment by aligning brain activity with the timing of sensory inputs [50]. Moreover, electrical rhythmic stimulation during sleep has been found to enhance neural entrainment and, as a result, to influence memory consolidation and brain plasticity [51]. Transcranial alternating current stimulation, which applies sinusoidal currents to ongoing brain oscillations, has been used to investigate neural entrainment and its role in characterizing brain function [52,53,54], whereas the entrainment of blood pressure and heart rate oscillations can be induced by periodic breathing activity [55]. Conversely, other cyclical and rhythmic physiological mechanisms (e.g., heart rate) have only a limited entrainment capacity to affect external rhythmic cues. Such limitations are believed to stem from evolutionary mechanisms that protectively regulate autonomic functions independently of external stimuli, ensuring the maintenance of homeostasis [56]. However, despite the presence of some autonomic physiological protective mechanisms, some studies showed a state of order, stability, and harmony in the oscillatory outputs of the regulatory systems, defined as physiological coherence, which is reflected in higher-order sine-wave-like heart rate variability patterns [39].

Research on temporal activity has primarily focused on the relationship with brain cortical activation in response to auditory stimulation through EEG and magnetoencephalographic techniques [57,58,59,60,61,62]. The EEG technique is widely used and is a particularly effective tool for measuring neural entrainment because of its high temporal resolution, which allows it to capture the precise timing of brain oscillations in response to external stimuli, as observed in research conducted by Nozaradan et al. [63], which aimed to tag neural entrainment according to the beat and meter of the stimuli. By analyzing EEG-evoked oscillations and modulation in response to rhythmically induced activity or stimulation, it is possible to gain a deeper understanding of the fundamental mechanisms of entrainment that can underpin cognitive processes, affective modulation, and motor coordination abilities [64,65,66].

**Figure 2:**
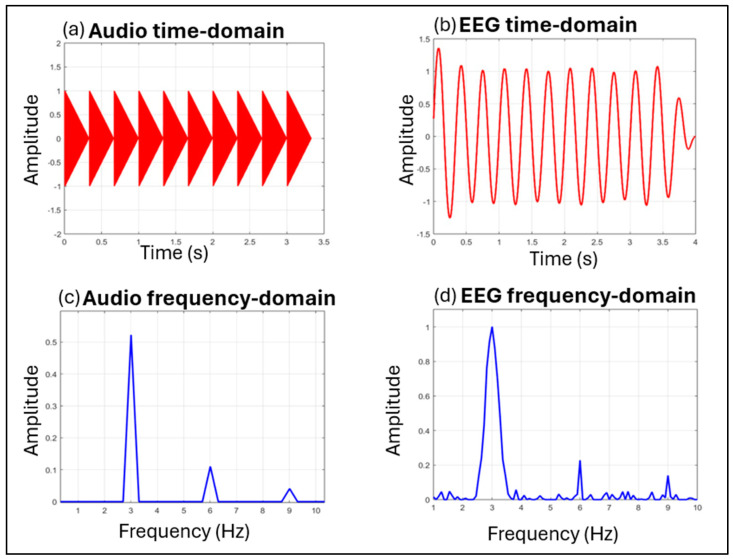
**EEG frequency-tagging method.** Panels (**a**) “Audio time-domain” and (**b**) “EEG time domain” display the time-domain representations of a 3-Hz auditory rhythm, while panels (**c**) “Audio frequency-domain” and (**d**) “EEG frequency-domain” display the frequency-domain representations of a 3-Hz auditory rhythm. The audio tone signal envelope in (**a**) illustrates a regular oscillation at 3 Hz, while (**c**) shows the corresponding audio peak of 3 Hz in the frequency spectrum. Panels (**b**) and (**d**) depict the EEG responses from a listener (simulated data for portrayal purposes) whose neural activity is entrained with this rhythmic input. In panel (**b**), the EEG trace reflects synchronized oscillations at 3 Hz, while (**d**) demonstrates a prominent 3-Hz peak in the neural frequency spectrum, confirming phase-locking between the auditory stimulus and brain oscillations. This approach exemplifies neural rhythm processing using the EEG frequency-tagging method, through which brain activity synchronizes to rhythmic stimuli. The method quantifies the degree of neural entrainment by examining peaks in the EEG spectrum, particularly at frequencies corresponding to rhythmic inputs.

One promising approach for investigating neural rhythm processing is the EEG frequency-tagging method [67,68] (Figure 2).

The EEG frequency-tagging method measures neural entrainment to rhythmic stimuli by detecting peaks in the EEG spectrum at frequencies corresponding to the rhythm envelope of auditory or visual stimuli [69,70]. The neural peaks, observed as steady-state evoked potentials (SS-EPs), represent the dynamic coupling of cortical activities with external stimuli and are specifically observed in response to beat and meter frequencies elicited under various conditions. For instance, SS-EPs are observed in the mental imagery of meter, spontaneous beat induction from rhythmic patterns, multisensory integration, and sensorimotor synchronization. Previous findings suggest that SS-EPs elicited in response to rhythmic stimuli do not simply reflect a direct encoding of the external rhythm; rather, the brain actively transforms the incoming rhythmic input by amplifying frequencies that align with a perceived beat or meter. This transformation highlights the brain’s capacity to emphasize rhythmic patterns that correspond to subjective perceptions of beat and meter [63]. Several studies have explored this phenomenon using frequency-tagging methods, demonstrating that SS-EPs elicited at frequencies corresponding to a perceived beat and meter are shaped not only by stimulus properties (bottom-up processing) but also by top-down mechanisms such as movement or predictive timing processing [71,72,73].

**Figure 3:**
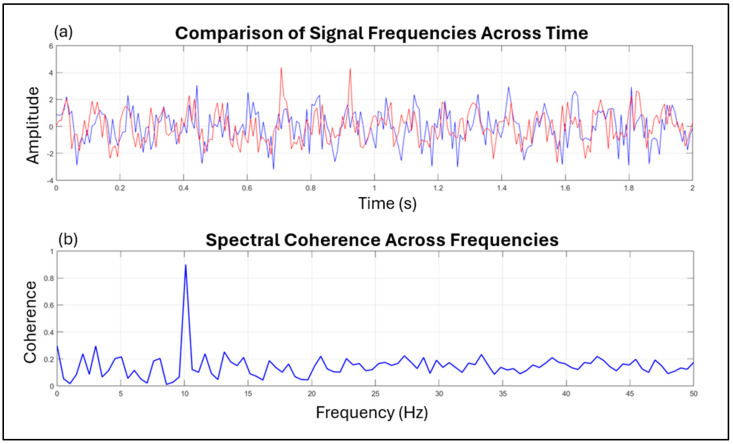
**Coherence index.** This figure depicts two graphs that compare two signals over time and shows how they are related to different frequencies. The upper panel (**a**) “Comparison of Signals Frequencies Across Time” displays both signals (red signal and blue signal) over a 2-s period. These signals fluctuate in a similar way and appear to share a common rhythm at around 10 Hz (i.e., 10 peaks per second). However, some random noise causes small differences between them, such as minor shifts in peaks and troughs. Overall, the two signals appear to follow a similar pattern, despite the noise. The bottom panel (**b**) “Spectral Coherence Across Frequencies” represents the coherence between signals, which measures the coupling of these signals at different frequencies. Coherence values range from 0 (no relationship) to 1 (a perfect match). There is a clear peak at around 10 Hz, showing that the two signals are highly related to this frequency. Beyond this, the coherence decreases, meaning that the signals do not align well at other frequencies. This suggests that their coupling is strongest at around 10 Hz, while, at other frequencies, they behave more independently. In summary, the two signals are most strongly related at around 10 Hz, which is clear from both their time-domain behavior and the high coherence at this frequency. Outside this range, their relationship weakens. This type of analysis is useful for understanding how signals relate and are coupled to each other within different frequency ranges, especially when noise is involved.

A common method to assess entrainment at the neural level, expressed as the phase-locking of neural responses to rhythmic stimuli, is the evaluation of the inter-trial phase coherence (ITPC) index [74,75]. This index (also referred to as inter-trial coherence or the phase-locking factor), quantifies event-related phase modulations. ITPC values range from 0 (indicating high variability in phase angles across trials) to 1 (wherein all trials exhibit identical phase angles; cf. Figure 3).

**Figure 4:**
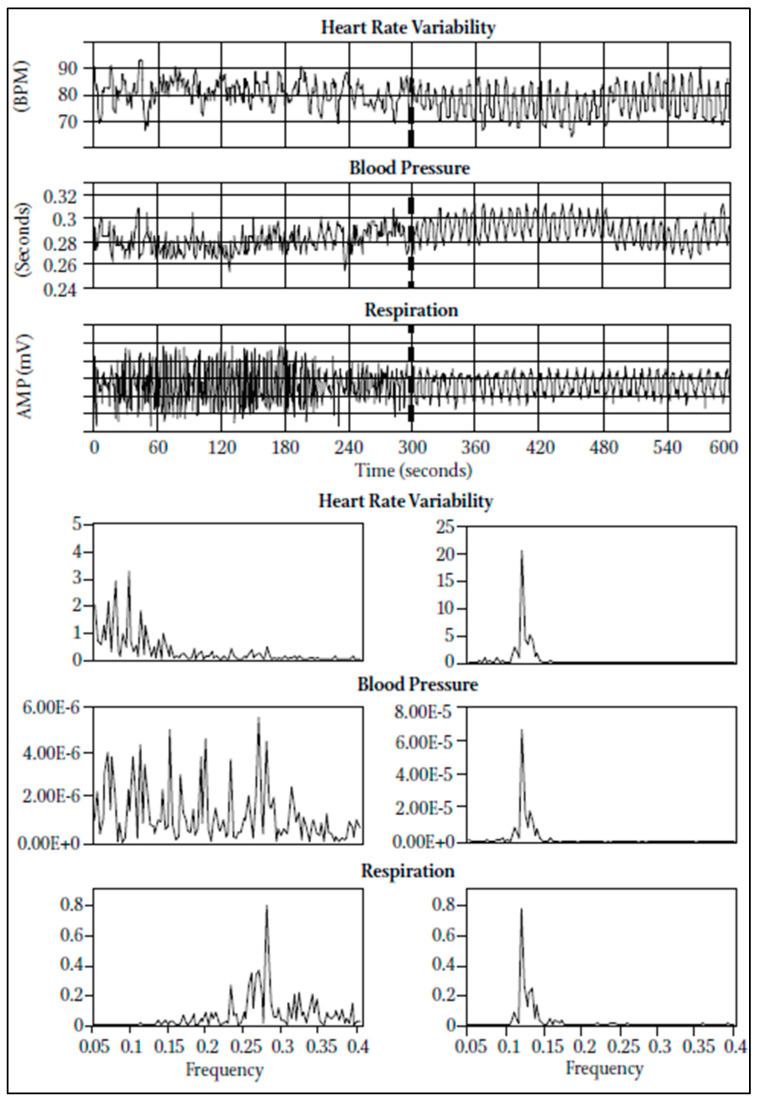
**Coherence and entrainment.** This figure shows the level of entrainment across three physiological systems of the human body: heart rate variability, blood pressure, and respiration rhythms over a 10-min period. Around the 300 s. mark, this participant engaged in the “freeze-frame positive emotion refocusing technique”, which is designed to induce a state of coherence. This technique aligns the physiological systems, bringing them into entrainment in such a way that their rhythmic cycles synchronize. The bottom graph panel depicts the frequency spectra before and after the entrainment process. The bottom-right graph marked with “Baseline” and “Entrainment” shows that heart rate variability, blood pressure, and respiration have synchronized with the same frequency (figure taken from Ref. [82], reproduced with the permission of the *HeartMath Institute*; https://www.heartmath.org/).

However, the findings show that apparent changes in the inter-trial phase-locking of oscillatory activity can occur independently of any actual change in the phase of ongoing activity, inviting caution when interpreting ITPC in cognitive neuroscience, as it may not accurately reflect cognitive processes like attention [75]. Interestingly, other techniques can be used to measure brain coherence. For example, some studies used near-infrared spectroscopy, a non-invasive method that uses light to monitor blood flow and oxygenation levels in the brain [76], to simultaneously measure brain activity in two people playing a computer-based cooperation game side by side [77], and in human dyads engaged in cooperative interactions [78]. Inter-brain activity coherence was calculated between participants, suggesting that coherence between the signals generated by their right superior frontal cortices increased during cooperation [77]. The concept of coherence (see Figure 4) is widely adopted in reference to biological systems, as the degree to which different physiological signals are synchronized and operate in harmony [79]. As previously discussed, in the context of brain activity, higher coherence between distinct regions of the brain suggests that these areas are functionally coupled. However, coherence, defined as an organized state of relatively sine-wave-like signals, is equally significant when examining heart rate rhythmicity [80]. This is particularly relevant since heart rate variability (HRV), a measure of the variation in time intervals between consecutive heartbeats, reflects autonomic nervous system regulation and has been associated with well-being [81]. Evidence suggests that when heart rate rhythms synchronize with respiratory cycles, this signals a state of coherence, indicating a balanced autonomic response [82]. Such synchronization can be observed during relaxation practices, such as meditation and deep breathing exercises or positive-emotion-focused techniques, which promote emotional regulation and parasympathetic activation, as better described by McCraty and Childre [83]. In their seminal work, developed at the HeartMath Institute, McCraty and Zayas [39] (p. 3) postulated that “physiological or cardiac coherence is reflected in a more ordered sine wave-like heart rhythm pattern, associated with increased vagally-mediated HRV, entrainment between respiratory, blood pressure, and heart rhythms, and increased synchronisation between various rhythms in the EEG and the cardiac cycle”.

Physiological or cardiac coherence emphasizes how the entrainment process can be associated with HRV patterns, which emerge during positive emotional experiences and relaxation, indicating optimal autonomic balance and improved physiological coordination across different body systems (e.g., blood pressure or respiration). The authors ultimately proposed a definition of coherent rhythms in heartbeats, whereby “coherence” is described as “a relatively harmonic (sine wave-like) signal with a very narrow, high-amplitude peak in the low frequency (LF) region of the HRV power spectrum with no major peaks in the Very Low Frequency (VLF) or high frequency (HF) regions. Coherence is assessed by identifying the maximum peak in the 0.04–0.26 Hz range of the HRV power spectrum, calculating the integral in a window 0.03 Hz wide centred on the highest peak in that region, and then calculating the total power of the entire spectrum. The coherence ratio is formulated as: [Peak Power/(Total Power − Peak Power)]” [82] (p. 14).

Entrainment is not just a process in which physiological rhythms tend to align with external stimuli; it is also a phenomenon observable at the behavioral level, which is measurable using different approaches and tasks. One of the most effective methods for evaluating motor entrainment is by means of sensorimotor synchronization tasks (for an extensive review, see Ref. [84]). Such tasks require the participants to move in time with a rhythmic stimulus using a body part (like the fingers, arms, or feet), which provides a measure of a person’s ability to perform rhythmic movements in synchrony with external cues. It is also interesting to note that synchronizing one’s movements with an external rhythm can take many forms, including moving one’s limbs to an auditory sequence, walking, dancing, or in the context of musical ensemble performance [85]. However, the most commonly used approach involves asking participants to produce regular, rhythmic taps with their fingers, which can briefly be explained as follows [86]. First of all, it is possible to distinguish at least two main tapping task paradigms, based on their functional structure. The first paradigm refers to unpaced tapping tasks. In these tasks, participants tap at a self-selected rate, a test that is often used to measure their spontaneous motor tempo, or how consistently they can maintain a steady tempo. In contrast, the second paradigm refers to paced tapping tasks, which require participants to synchronize their taps with an auditory cue, such as a steady sequence from a metronome tone. Here, the requirement is to match each tap with the beat or with a more complex stimulus, like music. When participants are asked to stop tapping as soon as the cue fades, the task is referred to as synchronization without continuation. If they must continue tapping at the same speed after the cue stops, this is called a synchronization-continuation task.

Variability and asynchronies (also known as synchronization errors) are typical metrics for measuring sensorimotor synchronization performance [84,85,87]. Asynchrony refers to how closely the taps align with the beat when synchronizing to an external metronome or rhythm. Taps that occur before the beat are negatively asynchronous, while taps after the beat are positively asynchronous. The standard deviation of asynchronies is also an important index of stability in synchronization. Alternatively, the mean and variability of inter-tap intervals (ITIs) are key measures of synchronization performance during continuation tapping tasks. While the mean ITI indicates whether a participant maintains or deviates from the original tempo, ITI variability reflects how consistently the taps are spaced. This variability is typically expressed as the standard deviation (SD) or coefficient of variation (CV; SD divided by the mean ITI). Sometimes, variability is described inversely as “consistency”. These metrics—mean ITI and ITI variability—are also used in self-paced tapping tasks, in which there is no external stimulus.

In this context, a study of particular interest was conducted by Rosso et al. [88], which provides an index of stability that serves as a measure of neural entrainment for auditory–motor synchronization. This index provides a global neural outcome measure associated with overall motor synchronization performance. More specifically, the Stability index is computed from EEG signals to extract the component that is maximally entrained to a periodic auditory stimulus. This involves analyzing frequency fluctuations over time, which reflects how consistently the neural responses align with auditory cues. This study hypothesizes that a stable performance in behavioral tasks, such as tapping in time with a metronome, will correlate with a stable entrained neural component, as measured by the stability index. In essence, a higher stability index indicates a stronger and more consistent coupling between auditory stimuli and motor responses, while greater fluctuations in this index suggest poorer synchronization and performance.

Notably, eye tracking—an experimental method that tracks eye movements and gaze positions across different tasks and time intervals [89]—has also been used to measure motor coordination systems, particularly in research on eye–hand coordination [90,91]. Interestingly, a study conducted by Schmidt et al. [92] highlights the role of eye movements during visual tracking as a pivotal factor in understanding how visual stimuli can influence motor coordination. This research suggests that movements provide important clues about the dynamic synchronization processes involved in the rhythmic entrainment process.

Furthermore, when measuring the entrainment phenomenon, it is crucial to clearly identify the specific functional mechanisms being investigated—such as the synchronization of neural oscillations supporting attentional processes, or motor responses to repetitive perceptual stimuli. Ensuring methodological rigor is critical, as this directly guides the selection of appropriate measurement techniques and defines the specific outcomes to be assessed. As previously discussed, entrainment can be associated with various temporal dynamics that support functional activity across cognitive, motor, affective, and social domains. In this context, it is essential to highlight that the oddball task paradigm can be an effective method for measuring the entrainment-related effect. Oddball tasks are specifically designed to introduce occasional “deviant” stimuli within a sequence of repeated, “standard” stimuli, to elicit an unexpected response to target stimuli, and can be adopted in an active paradigm (i.e., when participants respond to the target stimuli actively), or in a passive paradigm (i.e., when participants are not required to respond [93]). Auditory and visual oddball tasks, combined with EEG measurements, are widely employed in cognitive neuroscience to investigate how the brain detects and processes deviant stimuli, such as changes in the pitch or duration of auditory sequences or alterations in visual features.

EEG event-related potentials (ERPs), such as mismatch negativity (MMN), which reflects the brain’s automatic detection of unexpected deviations [94], and P300 components, which are associated with attentional resource allocation and working memory update [95], are typically investigated in response to deviant target stimuli. Combined with behavioral performance measures and specific manipulations of the target stimuli in different experimental conditions, this approach may offer a valuable assessment of how the brain processes entrainment dynamics in terms of “deviations”, i.e., violations of rhythmic expectations.

Neural, physiological, and behavioral assessments can potentially be used in comparative studies between healthy individuals and clinical populations. These methods are often adopted in research involving individuals with neurological disorders, whereby distinct components of cognitive, motor, or perceptual functions can be examined separately. For instance, cerebellar lesions are particularly significant, due to the cerebellum’s role in encoding precise event timing on the micro-timing scale [96,97,98]. In addition, research has shown that individuals with cerebellar lesions experience impaired neural entrainment regarding auditory rhythms, especially at faster tempos [99]. Future studies in this field could bridge the gap in our understanding of how motor and auditory systems interact during entrainment, potentially informing the development of innovative neurological rehabilitation strategies.

In this section, we have focused on the most relevant approaches to measuring physiological entrainment, with a particular emphasis on the role of physiological and behavioral assessments, as adopted in the most recent literature. Table 1 provides a comparative overview of the various measurement techniques described so far.

It is important to recognize that additional and combined techniques for measuring entrainment across various domains are also being employed. For instance, in the social domain, some approaches investigate either complex movement synchronization, as in dyadic and dynamic human interactions [100,101], or group rhythmic synchronization in the broader context of musical social entrainment [102]. Some of these approaches, along with key studies in the field, will be critically examined in the following sections, highlighting the role of entrainment in promoting mental health and well-being.

**Table 1:** **Comparative overview of measurement techniques for entrainment phenomena**. This table provides a comparison of the various techniques described in Section 3, outlining their key characteristics and applications in various research contexts. Each technique is briefly described, highlighting its core methodology in the description, and the specific empirical application contexts in which it is most reliable.

Technique	Description	Applications
Frequency Tagging (EEG) [70,71,72,73]	Measures neural entrainment by detecting peaks in the EEG spectrum that align with the frequencies of external rhythmic stimuli	Used for studying brain synchronisation to rhythms, especially in music, beat perception, and multisensory tasks. Useful for non-invasive studies with high signal-to-noise requirements
Inter-Trial Phase Coherence (EEG) [74,75]	Quantifies phase-locking of neural responses to rhythmic stimuli (values range from 0 to 1)	Facilitates the study of neural synchronisation with external rhythms, though variations in ITPC may not always correspond to underlying phase dynamics. Widely adopted in research on entrainment and subjective experiences such as groove or beat perception
Near-Infrared Spectroscopy (NIRS) [77,78]	Non-invasive technique monitoring blood flow and oxygenation levels to study inter-brain coherence	Adopted in studies on cooperative activities or dyadic interactions, where inter-brain activity coherence is of interest
Event-Related Potentials (ERPs) and Steady-State Evoked Potentials (SS-EPs)-(EEG) [66,72,93,94,95,103,104,105]	Investigates neural responses to deviant stimuli using ERPs components like MMN and P300 (often combined with oddball task paradigms), or to evaluate steady state evoked potentials (SS-EPs) elicited by sensory entrainment process	ERPs are reliable for assessing neural responses to deviations in rhythmic expectations and studying cognitive processes, such as temporal prediction, and working memory functioning. SS-EPs studies examine steady-state neural entrainment to perceived beats or meters, investigating the coupling between sensory and motor-related activities
Heart Rate Variability(HRV) [80,81,82,83]	Measures time interval variations between heartbeats to assess the autonomic nervous system balance	Used for investigating physiological coherence and relaxation-induced entrainment through breathing-based techniques or positive induced emotional states
Sensorimotor Synchronisation [84,85,86,87]	Assesses rhythmic motor coordination using asynchronies (known as synchronisation errors) and inter-tap interval (ITI) variability	Effective for evaluating motor entrainment tasks like tapping in synchrony with rhythmic cues; commonly used for motor and auditory rhythm interaction studies
Stability Index(EEG) [88]	Quantifies the consistency of neural entrainment to auditory stimuli using EEG, associated to behavioural performance in motor synchronization tasks	Useful for studying auditory-motor interaction and synchronisation. Can be applied in rehabilitation and motor coordination research, especially in linking neural signals to stable performance
Eye Tracking [89,90,91]	Tracks eye movement (e.g., saccades) to assess motor rhythmic coordination	Applied in studies of eye-hand coordination or visual tracking to understand synchronisation processes

## 4. Theories and Functions of Entrainment

Theories such as “Communication through Coherence” [40,106], “Communication through Resonance” [107], “Active Sensing” [108], the “Dynamic Attending” models [109,110,111], the “Predictive Coding” framework [112,113], and “Dynamic Information Selection by Entrainment” [21] attempt to explain the mechanism of entrainment by proposing that rhythmic synchronization in the brain facilitates efficient information processing and selective communication. Specifically, “Dynamic Information Selection by Entrainment” provides a unifying account of entrainment, suggesting that neural entrainment extends beyond the basic support of sensory information processing in the brain. This recent approach seeks to explain entrainment by integrating the model of network communication with sensory sampling mechanisms, emphasizing how the brain’s activity adapts to external inputs to facilitate cognitive processing and information exchange based on predictive models. In what follows, we will briefly outline the fundamental principles of these theories. Table 2 provides a comparative overview of the various theories and models of entrainment described in this section.

We have already mentioned the model known as Communication through Coherence (CTC) [40,106], which posits that coherent oscillations—such as phase-locked signals—can be independently generated in different brain regions. This theory highlights the role of neural synchronization in enabling effective communication between neural pools, allowing the brain to selectively process sensory information. According to CTC, different brain frequency bands (e.g., alpha, beta, gamma) can be associated with various cognitive functions, and the coherence within and between such bands influences how information is processed in the brain. For instance, in the context of attentional processing, it has been suggested that attended stimuli exhibit a stronger gamma-band synchronization effect [114]. In contrast, Communication through Resonance, as proposed by Hahn et al. [107], states that coherent oscillatory activity across brain regions is the direct result of the slow propagation of synchronous activity, which tends to promote its propagation along weak connections that would otherwise fail to propagate. Therefore, the coherence of brain oscillations is viewed as a natural outcome or a consequence of propagation dynamics, rather than as a specific instrumental requirement to establish communication through synchrony.

According to the Active Sensing model [108], entrainment allows the brain to synchronize with predictable sensory events by filtering irrelevant information out and enhancing the perception of relevant stimuli. This model prioritizes sensory inputs that temporally align with motor rhythms, ensuring efficient and selective processing. Attention plays a fundamental role in this process, as it underlies the neural rhythmic processing associated with motor activities such as sniffing, micro-saccades, and even low cortical oscillatory rhythms, which impose rhythmic patterns on the sensory inflow.

Interestingly, the mechanism of functional synchrony, attention and timing, has been investigated from different perspectives, which focus on the interplay between the predictive coding framework and dynamic systems models [115]. The Dynamics of Attending model emphasizes internal oscillations and periodic attentional activity [109,110,111], suggesting that internal oscillations drive individuals to synchronize their attention with external events. This theory highlights the role of expectancy in directing attention, which can be adjusted over time based on the context and responses to temporal expectations. The Predictive Coding model [112,113,116] posits instead that the brain continuously generates predictions about a sensory input, and updates such predictions based on actual experiences. This approach highlights how attentional processes are not only driven by internal rhythms but are also shaped to minimize prediction errors in response to external stimuli. This framework is strongly supported by a pioneering study on visual processing conducted by Rao and Ballard [117], which serves as a foundational contribution to the field. This study describes a hierarchical predictive coding mechanism on visual processing, in which the higher cortical areas generate predictions about incoming sensory information, while lower areas convey only the residual errors between predictions and the actual sensory input. Hence, the dynamic interaction between top-down predictions and bottom-up error signals allows the brain to efficiently process visual information by continuously refining its internal models to better align with the external world.

Recently, a new unifying framework of entrainment, known as Dynamic Information Selection by Entrainment (DISE), has been proposed as a dynamic entrainment model explaining how neural oscillations adapt in real-time to external or internal stimuli, prioritizing task-relevant information through phase alignment and flexible sensory integration [21]. This framework builds on existing theories of entrainment, highlighting the roles of environmental and bodily factors. The core principle of this model, in contrast to the concept of “network resonance”, is that entrainment primarily works as a dynamic selection mechanism for information. This perspective explains how different brain networks phase-align with expected stimuli, optimizing the information flow based on task relevance and predictive models. The mechanism of entrainment, as described in this model, involves “phase resetting”, whereby ongoing neural oscillations are modulated by external inputs. According to this model, neural entrainment is considered supramodal. This means that oscillations in one modality can be entrained by rhythmic inputs from different modalities (e.g., in communication, where visual cues can aid auditory perception). Moreover, in the DISE model, neural entrainment is described as an “oblique” process, suggesting that it can be triggered by various rhythmic sensory inputs, including environmental stimuli, self-generated actions (e.g., saccades or sniffing), autonomic processes (e.g., breathing), and top-down signals that facilitate internal information transfer. The authors also propose that while entrainment can occur automatically, it is primarily controlled by top-down processes and is not strictly “frequency-specific”, meaning that it can occur across a wide range of brain frequencies, including the delta, theta, alpha, and beta activity bands.

**Table 2:** **Comparative overview of entrainment theories and models.** This table summarizes the theories and models presented in Section 4, outlining their description, empirical support, predictive or descriptive nature, key terms, and similarities/differences with other theories/models. It compares the following theories related to the entrainment phenomenon: communication through coherence (CTC), emphasizing neural synchronization; communication through resonance (CTR), focusing on neural signal amplification; active sensing (AS), for sensory-motor prioritization; dynamic attending (DA), for temporal coherence; predictive coding (PC), for error minimization; and dynamic information selection by entrainment (DISE), for top-down rhythmic information integration.

Theory/model	Description	Empirical support	Predictive/descriptive	Key terms	Similarities and differences
Communication Through Coherence (**CTC**) [40,106]	Synchronised oscillations, enhance selective and effective neural communication	Gamma-band coherence with attentional selection, enhance connectivity and synchronisation during selective attention tasks	Descriptive; neural synchronisation as communication through "coherence". Needs further empirical support for predictive neural mechanisms	Neural synchronisation, coherence, selective attention	Aligns with PC on hierarchical processing, but focuses more on oscillatory coherence. Differs from CTR by minimising the role of propagation dynamics in coherence
Communication Through Resonance (**CTR**) [107]	Resonance amplifies weak neural synchronous activity, across cortical regions	Resonance supports the propagation of weak signals across feedforward networks, with oscillatory activity sustaining coherence without strong synaptic connections	Descriptive; emphasises how signals propagate through neural networks, rather than testing predictions about processes beyond signal amplification	Resonance dynamics, weak connection, feedforward networks	Shares the concept of coherence with CTC, though it emphasises resonance dynamics over independent oscillatory coherence. Differs from AS by giving less importance to motor rhythms in sensory processing
Active Sensing (**AS**) [108]	Motor rhythms entrain sensory pathways, by aligning sensory inflow, with task-relevant motor and attentional routines	Rhythmic motor activities (e.g., sniffing, saccades) synchronise sensory oscillations and amplify relevant inputs, suppressing irrelevant ones	Predictive; postulates sensory prioritisation and motor-sensory coupling mechanisms for perception	Motor-sensory integration, attention modulation, efference copy	Aligns with DA on the importance of rhythmic patterns in attention. Differs from PC, giving less emphasis to error-driven processing to perception
Dynamic Attending (**DA**) [109,110,111]	Coherent events enhance temporal predictions and anticipatory responses, while less coherent events demand analytic attending focused on local details	Role of temporal coherence in driving accurate time judgments and anticipatory behaviours in rhythmic contexts (e.g., music, speech)	Predictive; integrates temporal structure into attentional mechanisms, highlighting the role of rhythmic patterns in triggering attention	Temporal coherence, temporal predictability, event time	Aligns with AS on the importance of rhythmic patterns in attention. Differs from CTC by focusing on temporal structures rather than neural oscillatory coherence mechanisms
Predictive Coding (**PC**) [112,113,117]	Prediction errors dynamically interact to minimise error signals, optimising sensory processing and learning	Neural connections prioritise relevant information and uses precise error signals to refine and update its predictions	Predictive/explanatory; integrates perceptual inference, learning, and attention through error minimisation	Error signal, prediction error, efficient coding, hierarchical processing	Shares hierarchical processing principles with CTC, but focuses on error-driven updates rather than coherence. Differs from DA on the idea that temporal structure is the main driver of attention
Dynamic Information Selection by Entrainment (**DISE**) [21]	Entrainment operates as a dynamic selection mechanism that optimises task-relevant information	Relevance of supramodal entrainment, phase resetting, and multimodal integration, emphasising the entrainment top-down mechanism of control	Predictive; provides a unifying framework integrating sensory information gathering, dynamic filtering, and neural rhythm adjustments to external patterns	phase modulation, neural entrainment, rhythmic inputs	Shares PC’s focus on top-down modulation and supramodal integration. Contrast with CTR by considering entrainment primarily as a dynamic selection mechanism for information

## 5. Physiological Foundations of Brain Oscillatory Activity Across EEG Frequency Bands

As discussed above, entrainment can underlie several cognitive functions, especially at the neural level. A widely used approach to investigate the neural entrainment phenomenon is to examine the EEG frequency bands, which reflect rhythmic brain activity. These bands—delta (1–4 Hz), theta (4–7 Hz), alpha (8–13 Hz), beta (13–30 Hz), and gamma (30–100 Hz)—are generated by synchronized postsynaptic potentials in the pyramidal neurons [118].

As previously discussed, one of the most widely accepted models of brain oscillatory activity suggests that rhythmic brain patterns can directly reflect the predictive information processing involved in selective communication and information transmission [106,119]. However, the power of brainwave band activity and the coupling between brain oscillations continuously change in response to task demands [120]. In this regard, the literature suggests that neural entrainment activity operates according to the principles of nonlinear dynamics [121,122,123]. This model suggests that two rhythmic systems can achieve phase synchronization by aligning their cycles through mutual adjustment, driven by complex and dynamic interactions within the neural systems that modulate brain responses according to external stimuli processing and internal physiological states.

Among the brain’s band rhythms, neural entrainment in the beta band has been associated with several cognitive and motor functions, including visual perception and the imagery of biological movements [124,125]. Specifically, increased levels of beta oscillations in the prefrontal cortex have been implicated in working memory, decision-making, and cognitive control beyond sensorimotor control [126,127]. Moreover, a study conducted by Fujioka et al. [128] showed that beta-band oscillations in the brain represent both auditory beats and their hierarchical structure, such as in the march and waltz meters, by modulating beta-power in the auditory and sensorimotor regions during both the perception and mental imagery of musical rhythms. These findings highlight the role of beta oscillations in coordinating timing information and auditory–motor integration. Another experiment by the same group [129] aimed to examine the role of beta and gamma oscillations in the auditory cortices during passive listening to a regular musical beat with some tones omitted. Their results demonstrate that beta activity decreased after each tone and then increased, forming a periodic modulation that was synchronized with the stimulus, which the authors suggest reflects the exogenous processes related to auditory–motor communication. In contrast, gamma activity peaked following both tones and omissions, indicating its role in the endogenous anticipatory mechanisms involved in musical beat encoding.

Alpha oscillations have been proposed to influence both spatial and temporal visual attention, as well as attentional and cognitive efficiency. Specifically, increases in alpha power and phase entrainment have been observed in the hemisphere processing spatially unattended information [130], indicating that neural activity in this frequency band may serve as a reliable marker of visual system functionality [131,132,133]. In this regard, the frequency, power, and phase of alpha oscillations have also been associated with cognitive performance and timing, according to the inhibition-timing hypothesis [134]. In contrast, active processing in task-engaged areas is marked by synchronization in the gamma band, along with a reduction in alpha activity. Therefore, alpha activity is seen as a key mechanism for modulating attentional and cognitive efficiency by selectively inhibiting non-essential regions during task performance [135]. In the context of neural rhythmic activity within the alpha band, mu rhythms, which originate from the primary motor cortex in the 8–13 Hz range [136], are well-recognized indicators of motor system activity [137]. These rhythms reflect cortical idling, which refers to a state of the brain in which there is minimal or no active processing of sensory or cognitive information. Specifically, increased mu power—referred to as event-related synchronization—is observed during movement suppression, while decreased power—known as event-related desynchronization—occurs during active movements [138]. Research also showed that the mu rhythm can be a potential biomarker of empathic mimicry [139].

Theta and delta waves are also key players in rhythmic entrainment and cognitive functioning. For instance, working memory performance is sensitive to the phase and rhythm of externally applied stimulations. Researchers also identified theta/gamma phase-amplitude coupling as the most prominent form of cross-frequency coupling associated with working memory processing [140]. In this regard, it has also been shown that gamma networks desynchronize and theta networks synchronize during encoding and retrieval [140].

## 6. Entrainment in Cognitive, Motor, and Affective Functioning: Implications for Well-Being and Therapeutic Approaches

In recent years, the concept of entrainment has gained attention, not only for its role in characterizing basic neurophysiological processes but also for its potential to promote mental health and well-being [141]. Indeed, entrainment can influence and be involved in several processes such as perception, attention, working memory, language processing, motor control and coordination, empathy, social interactions, and temporal music skills [34,142,143,144].

Moreover, rhythmic music-based activities, which can effectively elicit entrainment between bodily systems, were adopted in rehabilitative, educational, and clinical research to promote cognitive and motor rehabilitation, as well as emotional regulation and social engagement [65,143]. In this section, we explore the impact of entrainment on cognitive, motor, and affective functioning, particularly in musical contexts, highlighting its potential applications in rehabilitative settings. Additionally, we discuss the impact of factors such as age, gender, and cultural differences in studies investigating physiological entrainment functioning.

### 6.1. Cognitive Functioning

In the context of auditory perception, brain rhythms synchronize with external auditory stimuli, enhancing the detection and prediction of temporal patterns [145]. This effect is particularly evident in the “cocktail party” effect, where the low-level auditory cortices are more responsive to attended vs. ignored speech, and only attended speech is tracked in the higher-order brain regions [146].

Furthermore, cognitive research has extensively investigated neural entrainment to understand attentional control mechanisms, suggesting that the neural basis of attention is inherently rhythmic [64]. The entrainment process has also been associated with attentional selection, as specifically demonstrated in a study conducted by Lakatos et al. [27]. Their study shows that aligning neural oscillations with rhythmic stimuli enhances attention by synchronizing the neural responses to task-relevant inputs, improving response efficiency. Thus, synchronization also implies faster reaction times, suggesting that attention is more effectively maintained when stimuli follow a rhythmic pattern. In addition, the results of this study show that the phase of low-frequency oscillations, such as delta waves, influences higher-frequency brain activity. Such brain-wave interaction underscores the relevance of timing in optimizing cognitive processing during attentionally demanding tasks.

Evidence also suggests that neural entrainment is a process underlying speech and language processing [147]. Although speech is continuous, meaningful units (such as syllables) are communicated at specific rates, a phenomenon that is particularly evident with syllables, which are typically produced at a mean rate of about five per second [12,148,149]. A consistent rate, such as a steady pace, creates a predictable rhythm for communication between speakers and listeners. This predictability allows the listener to anticipate the timing of the upcoming syllables, creating a temporal synchronization between their predictive models and the speaker’s speech patterns. Research indicates that the temporal structure of speech is essential, both for maintaining intelligibility [150] and for stabilizing communication, especially under challenging listening conditions [151]. Overall, neural entrainment may underlie the complex processes by which we perceive and interpret the meaning of words, phrases, and sentences [152]. Such processes are seen as forms of predictive action, whereby the listener not only perceives but also prepares for future speech, optimizing both the reception and the production of language [153]. The predictive mechanism of language is further supported by the bidirectional coupling of motor and auditory signals phase-locked to the rhythm of speech [21].

In this context, temporal processing is recognized as the main component of cognitive mechanisms, such as language, one that also underpins predictive behaviors [154]. Predictive mechanisms in cognitive research are widely examined using event-related potentials (ERPs), such as mismatch negativity (MMN) [94] and P300 components [95], often in oddball task paradigms (see Section 3). Some studies suggest that MMN can be used in basic research to investigate the neural signature of sensory prediction, specifically regarding how the brain detects and updates perceptual information according to the mechanism of adaptation and deviance detection in sequences of rhythmic and predictable stimuli [94,155,156,157,158]. Regarding P300, other studies have explored how transcranial alternating current stimulation, which modulates oscillatory entrainment activity, can affect the amplitude and latency of the P300 wave [159]. Similarly, another study suggests that by combining 40 Hz, flickering, with cognitive tasks during the entrainment stimulation, it is possible to evoke stronger neural responses in P300 for both amplitude and latency [160]. These findings highlight how ERPs can be usefully adopted to investigate the neural mechanisms involved in the predictive and adaptive functioning of the neural entrainment process.

### 6.2. Rhythm and Motor Well-Being

Research indicates that the motor system is highly sensitive to auditory priming and temporal stimulation [56]. For example, auditory stimuli can entrain motor responses, leading to improved performance in tasks requiring precise timing and movement synchronization [34,84]. Evidence shows that rhythmic auditory cues, such as a metronome beat, can prime motor synchronization by enabling finger and arm movements to align with the stimulus tempo and maintain synchronization, even when subtle, imperceptible tempo changes are introduced [161]. Moreover, under appropriate conditions, neural entrainment can reduce cognitive effort and attentional demands, improving both cognitive and motor functioning [162].

In this regard, the steady-state evoked potential (SS-EP) is a common EEG neural signature that is used to investigate the response elicited by the sensory entrainment process. This event-potential measure consists of continuous, frequency-specific neural responses generated by the brain in response to periodic sensory stimuli, such as rhythmic sounds [163] or flickering lights [164]. SS-EPs allow researchers to assess neural processing and brain activity and can be used to target a broad array of cognitive and neural processes [103].

Relevant studies were conducted to investigate neural entrainment by applying SS-EPs. For instance, Nozaradan et al. [72] conducted a study combining a tapping task with an EEG to analyze SS-EPs and investigate the neural mechanisms underlying sensorimotor synchronization. Their findings suggest that rhythmic sensorimotor synchronization relies on a dynamic coupling between sensory and motor-related activities.

A similar study design was applied by Colling et al. [66] to a group of children with developmental dyslexia, revealing atypical SS-EP patterns. These results indicated significant differences in neural rhythmic entrainment during both passive auditory listening and sensorimotor coupling conditions, suggesting deficits in auditory–motor integration. These findings highlight how SS-EPs can be adopted to investigate neural mechanisms in tasks requiring precise temporal coordination.

Research has shown that music training, which often involves rhythmic elements, promotes the far transfer effect, i.e., the strengthening of cognitive, emotional, social, and motor skills through music-based training interventions [104,143], which, in turn, can affect neural oscillations [105,165]. These studies show that the temporal and sequential nature of sound in music may serve as a “scaffold” that helps the brain develop the ability to represent and process temporal sequences in cognitive functions [105], and that motor rhythm can assist the motor networks in such a way that organized motor skills can be performed automatically and flexibly [165]. Therefore, auditory stimuli may be relevant cues for temporal ordering and information sequencing [166], and can be involved in learning and memory processing [167]. Interestingly, a recent study by Albouy et al. [168] demonstrated that rhythmic visual stimulation can enhance frontoparietal connectivity and causally improve auditory working memory performance [168]. These results were also confirmed by a recent randomized controlled trial study conducted by Nuernberger et al. [169]. Their results showed that intense visual stimulation can enhance visuomotor integration, improving motor learning performance and visual short-term memory, supporting the supramodal nature of the entrainment phenomenon.

### 6.3. Music and Affective Well-Being

Beyond enhancing cognitive functions and motor coordination, rhythmic entrainment has been shown to strengthen interpersonal connections by fostering affiliation and intimacy during social interactions [170]. These effects are particularly evident in shared musical experiences [1,171,172], wherein synchronized movements promote emotional sharing, support empathic connections, encourage group bonding [173], and nurture prosocial behavior [65]. When the body actively engages in perceiving and interacting with music, as described in the embodied music cognition framework [172], the rhythmic alignment between the listener and the music stimulus reflects a dynamic interplay and continuous adaptation between physical movement and musical perception [174]. This interaction enables the body to synchronize its movements with rhythmic inputs, reducing the prediction error—defined as the discrepancy or as a gap between the expected and actual sensory input—while enhancing the overall musical experience [175].

Musical activities can contribute to shaping brain networks by converting auditory stimuli into motor representations, enhancing audio-motor integration, engaging perceptual, motor, and cognitive domains, and activating the mirror neuron system [176]. Evidence suggests that musicians exhibit greater mirror neuron activation when listening to music performed with expressive “enjoyment” and this activation is moderated by their level of musical expertise [177]. Such evidence aligns with the simulation theory of empathy, which proposes that motor processing plays a role in empathy—especially in spontaneous movement to music—by enabling individuals to model the actions of others [178,179,180].

Mirror neurons are thought to be a key signature of empathy [181]. Empathy is a fundamental process that allows individuals to quickly and intuitively resonate with the emotional states of others, and it is a critical component involved in regulating social interactions [182]. A relevant aspect of empathy involves unconscious emotional mimicry, which leads to affective sharing between oneself and others [183,184,185]. Such affective sharing is driven by the perception–action coupling mechanism, underpinned by the mirror neuron system, which automatically activates sensorimotor representations and responses in the observer [186]. In this context, mu rhythm suppression, has been associated with mirror neuron activity [187,188,189,190]. Mu suppression serves as an indicator of sensorimotor engagement, particularly when individuals are observing others in painful situations, further emphasizing mu rhythms implication in empathy-related processes [191].

Additionally, the entrainment process underlies both “physical mirroring”, such as gestural mimicry in communication or dance, and “metaphorical mirroring”, as observed in empathy, where individuals emotionally resonate with and understand the experiences of others [192,193,194]. Spatiotemporal mirroring is also evident in the bond between parents and infants, often observed through their synchronized gestures and vocalizations [195,196,197,198,199].

Moreover, Trost et al. [65], proposed a model of entrainment associated with music affect induction mechanisms that occur on social, motor, perceptual, and autonomic levels. This modelsuggests the potential of entrainment implications for affective processes such as empathy within the musical context [139]. In addition, Trost et al. [65] propose that the basal ganglia may play a key role in connecting rhythmic entrainment to emotional responses. Through these processes, Trost and colleagues conclude that music fosters emotionally rewarding resonance and social bonding, thereby promoting positive emotional states.

This perspective aligns with the model of physiological coherence observed in heart rate variability, as proposed by McCraty et al. [38,39,82,200]. These studies suggest that positive emotions, such as appreciation and compassion, are associated with more coherent (i.e., more synchronized) HRV patterns, while negative emotions correlate with incoherent patterns. Positive emotions may also enhance physiological restoration. In fact, one study by McCraty et al. [201] indicates increased HRV coherence after participating in emotional self-management programs, suggesting the importance of HRV analysis in emotional risk prevention [202].

Regarding affect induction mechanisms of music listening, the BRECVEMA framework [203] identifies eight psychological mechanisms—brainstem reflexes, rhythmic entrainment, evaluative conditioning, emotional contagion, visual imagery, episodic memory, musical expectancy, and aesthetic judgment—that can explain how music induces emotions in listeners, highlighting the role of synchronized bodily rhythms with musical rhythms in terms of generating emotional responses. Another research study conducted by Juslin et al. [204] supports the idea that emotional responses to music are influenced by the way that internal bodily rhythms, such as respiration, synchronize with the external rhythm of the music and evoke emotional reactions. The authors also suggest that positive emotional experiences can activate the brain’s reward system, enhancing memory formation and promoting brain plasticity. This idea highlights how external rhythms, like music, can influence physiological rhythms, fostering a sense of connection between the listener and the music. A similar concept was proposed by Scherer and Coutinho [205], who suggested an integrated framework that links the perception and cognition of music to the production of emotions through various psychobiological pathways, including entrainment. Therefore, as the rhythmic structure of music aligns with attentional fluctuations, emotional responses may influence the attentional focus by either enhancing or redirecting attentional focus during task engagement [206,207].

### 6.4. Applications for Well-Being

Regarding the application of entrainment to dyadic settings, Carlson et al. [142] recently conducted research in a non-clinical cohort, showing that synchronized movement plays a key role in enhancing emotional connection and empathy between participants. Similarly, a study conducted with 8- to 9-year-old children demonstrated that synchronized movement experiences promote cooperation and closeness among individuals. The results showed that children who engaged in synchronous rhythmic interactions perceived their partners as more similar and closer to themselves compared to those under asynchronous or non-interactive conditions [208]. A study by Wessler et al. [209], investigating movement synchrony in therapeutic settings with patients diagnosed with depression, suggested that entrainment through synchronized movement fosters stronger emotional bonds and improves responsiveness, creating a more supportive therapeutic environment. Therefore, their results showed that increased synchronized movements in therapy are associated with positive treatment outcomes. In addition, other studies have highlighted the benefits of dancing on well-being, particularly in non-professional adults, suggesting that shared rhythmic experiences not only promote physical and emotional health but also strengthen social connections, fostering a sense of community and enhancing interpersonal bonds [210]. Furthermore, research involving young healthy adults aged 18–36 years (equally distributed between males and females), underscores the role of interpersonal synchrony in social perception [211]. Findings from simulated dyadic interactions revealed that stable forms of coordination, such as in-phase and anti-phase synchrony, elicited the highest judgments of bond and affectiveness, expressed in terms of “rapport”, regardless of whether synchronization was conveyed through visual or auditory cues. These findings suggest that entrainment could play a relevant role in different clinical and non-clinical settings, not only by enhancing emotional bonding and empathy but also by supporting well-being across different age-group samples. Additionally, the simulation theory of empathy suggests that the audio–motor integration of the human mirror neuron system can be supported by music-based interventions in rehabilitation [176]. One example of music group therapy is the music embodied approach based on the “Dalcroze Eurhythmics” methodology [212] that explores musical elements through an awareness of body movements. This approach was adopted in clinical research and was shown to improve dynamic motor ability and cognitive function in clinical populations, such as older adults [213,214], children with attention-deficit hyperactivity disorder (ADHD) symptoms [215], and children with autism spectrum disorder (ASD) [216]. The Dalcroze methodology emphasizes the integration of rhythmic movement, as a product of musical expression and sensory–motor engagement, to enhance both cognitive and emotional development [217]. By promoting synchronized movement, music-based interventions can encourage emotional connection and cooperation, while also enhancing cognitive functions such as attention, memory, and motor coordination [218].

As regards rhythmic motor improvement, “Rhythmic Auditory Stimulation” (RAS) is a widely-known music therapy technique that uses rhythmic cues, such as metronome beats or structured music, to support the rehabilitation of rhythmic movements, particularly in individuals with Parkinson’s disease [219,220]. Such rhythmic cues are individually matched to each patient’s gait cadence and gradually adjusted to promote rhythmic entrainment. For example, in a study involving hemiparetic stroke patients, matched by gender, age, and lesion site, RAS was shown to significantly enhance gait velocity and stride length, and to reduce variability in muscle activation compared to conventional therapy, highlighting its potential application in motor rehabilitation [221].

Other rhythm-based music interventions include “Patterned Sensory Enhancement”, which uses rhythmic and melodic cues to regulate movement patterns, and “Therapeutic Instrumental Music Performance” (TIMP), which involves playing instruments, such as keyboards, strategically positioned to target specific motor functions [222,223]. Such interventions are widely recognized for their effectiveness, particularly TIMP therapy, in enhancing motor skills and supporting therapeutic outcomes in clinical conditions. For example, the TIMP approach was tested by Karatekin and Icagasioglu [224]. In their study, TIMP was employed with nine adolescent patients with cerebral palsy (five spastic hemiplegic and four spastic diplegic patients) and nine healthy adolescent controls, through piano training sessions twice a week for three months. The results showed significant improvements in grip strength and finger strength (notably in the fourth and fifth fingers) and in gross and fine motor skills. Moreover, a recent study [225] confirmed the benefits of TIMP in a group of adults with chronic post-stroke hemiparesis. In this randomized controlled trial study conducted on 30 participants (aged 33–76), TIMP significantly improved paretic arm control, and a similar effect was observed on the motor imagery condition.

Neuromotor rehabilitation techniques also integrate the principles of music therapy, as already evidenced in several neurological applications [56,226], particularly for individuals with movement disorders [34]. For example, rhythmic motor rehabilitation was recently applied to Alzheimer’s disease [227], Parkinson’s disease [220], stroke rehabilitation [228,229], traumatic brain injury [230], and cerebral palsy [224,231]. Such an approach highlights the potential of rhythm-based interventions to enhance motor function and recovery [232].

Evidence suggests that entrainment through music supports mental health and well-being in both clinical and non-clinical populations, fostering body integration and regulating physiological and emotional responses [233], especially through coordinated rhythmic motor movements [34,102,144]. For instance, the alignment of motor and sensory rhythms at the neural level facilitates the perception of motor actions—as observed in finger-tapping tasks [234]—and improves gait synchronization, as widely reported in clinical settings [235]. Interestingly, a study conducted by Dotov et al. [235] found that auditory–motor coupling was enhanced through interactive stimuli in both individuals with movement disorders, such as Parkinson’s disease, and a healthy control group. The results indicated that a strong entrainment effect was observed in clinical patients and healthy participants, with no significant differences between groups. This evidence suggests that interactive, adaptive stimuli are equally effective in supporting motor synchronization in both clinical and healthy populations.

As observed, entrainment is also a natural process that is involved in speech processing, which consists of the alignment of neural synchronization with the auditory speech envelope [236]. Some studies suggest that cortical entrainment offers a useful means of capturing speech processing differences, especially in elderly hearing-impaired adults, or in those with neurodevelopmental disorders [237]. However, a study on typically developing children aged 5 to 14 years [238] found that they did not adjust their speech rate to match the dynamic rate of a virtual interlocutor’s speech. This result suggests that the ability to synchronize speech rate with an interlocutor develops with age, highlighting the need to understand how speech entrainment progresses during typical development. Nevertheless, the beneficial effects of speech entrainment therapies are well-documented in clinical adult populations, for samples of men and women alike [239,240]. Speech entrainment therapy (SET) is an example of entrainment therapy, structured as a computerized clinical technique that involves mimicking an audiovisual speech model to enhance speech production. SET has proven effective in improving speech fluency in individuals with non-fluent aphasia [239,241], highlighting its therapeutic potential in speech rehabilitation.

Moreover, studies that applied binaural beats to non-clinical samples have shown that binaural stimulation can modulate brainwave activity and reduce stress, as evidenced by restoring alpha asymmetry in non-clinical populations, as well as by restoring partial alpha in stressed individuals [242]. Additionally, a significant enhancement of working memory capacity has been observed in young university students after binaural stimulation [243]. However, the duration of the brainwave rhythmic stimulation can influence the extent of such improvements, highlighting a need for further research in this area [244].

While there are several sources of evidence discussing entrainment as a relevant physiological process, there is less evidence explicitly addressing cultural differences in entrainment physiological studies. However, ethnographic research models [245] highlight how cultural differences influence the entrainment process by integrating diverse musical traditions, social contexts, attention dynamics, and individual factors such as tempo preferences, cognitive strategies, and emotional connections. In line with this model, Kaplan et al. [246] showed that cultural differences can impact entrainment, particularly how individuals perceive and synchronize with rhythmic patterns, shaping the listeners’ expectations. The same study also presents a model called “Phase Inference from Point Process Event Timing” (PIPPET), suggesting that cultural variations in rhythm perception arise from an inference process where listeners rely on learned musical patterns to predict and synchronize with rhythmic stimuli. This model finds support in comparative studies. For example, a study conducted by Kirschner and Ilari [247] investigated how Brazilian and German preschool children learned to synchronize movements with sounds through social interactions. Using joint drumming tasks with an experimenter—who was either visible or hidden behind a curtain—or drumming along with a playback beat, the study found that Brazilian children were more likely than German children to spontaneously synchronize their drumming. This difference was partially attributed to variations in individual experience with active musical practice.

## 7. Conclusions

In this review, after discussing the different terminological inconsistencies and empirical methodologies related to entrainment measures, we examined the role of physiological entrainment as a unified framework for cognitive, motor, and affective functioning, its role in information processing across different bodily systems, and its implications for well-being.

As we examined in detail, the phenomenon of physiological entrainment appears to be a ubiquitous process that manifests in various forms, including synchronization through interactions between single cells, heart rate, respiration, neural oscillations, motor coordination and social interaction [21,34,54,248].

We discussed the relevance of physiological entrainment as a fundamental mechanism that underlies effective network communication functioning [106,107,108,119]. In this regard, we explored how neural entrainment enhances task-relevant information processing, discussing recent theories that emphasize the relevance of the neural entrainment phenomenon to cognitive functions and interactions with the environment [21]. We then discussed how the rhythmic structure of environmental stimuli allows temporal mechanisms in the brain to synchronize with these patterns by facilitating more efficient network processing and enhancing its predictive capabilities [112,113].

We also examined the significance of the entrainment process for various cognitive functions [34,142,143,144], especially those concerning language processing, explained as a dynamic dyadic entrainment interaction [37,174,249]. We showed the relevant mechanism by which the temporal structure of speech can align with rhythmic stimuli, supporting speech-related functions [150]. Such temporal synchronization is not merely a byproduct of communication; rather, it serves as a vital component that facilitates the attribution of meaning to sensory information [249].

In this context, we emphasized how entrainment extends across auditory–motor processing systems, supporting coordination and synchronization between auditory and motor functions in response to rhythmic stimuli [230,250]. While the motor cortex is actively involved in speech processing, the auditory cortex is thought to provide feedback about incoming sounds [24,25]. In this regard, research on entrainment indicates that the motor system remains highly sensitive to auditory priming and temporal stimulation [56], as demonstrated by sensorimotor activity, wherein rhythmic auditory cues can prime motor synchronization, enhancing timing accuracy and motor coordination, as observed in musical expertise [34,142,143,144], suggesting the potential implications of rhythmic motor-based interventions [232].

We investigated the potential implication of physiological entrainment as an affect–induction mechanism through music [65], its implication for well-being, as expressed by HRV coherence states [39,82], and its implication for empathy [139]. In this context, at the neural level, mu rhythms were discussed as a potential biomarker of empathic mimicry [139]. As regards the behavioral and social levels, we observed that entrainment can enhance the ability to engage in joint actions and social interactions, with possible therapeutic interventions supporting well-being [34,174].

Notably, we also observed how the terminology in the literature on “entrainment” is often inconsistent, underscoring the complexity and multidimensional nature of this phenomenon. In fact, research often adopts different terms and theoretical frameworks, leading to potential misunderstandings in the field. Therefore, we believe that establishing a unified framework, as recently proposed in the model of entrainment by Lakatos et al. [21], along with consistent terminology, is an essential step for advancing research in this area. To address this issue, we decided to adopt “physiological entrainment” as the main key-term, to designate the primary process of synchronization between bodily rhythms and external rhythmic stimuli. In the framework of embodied cognition [32,175], such a definition conceptualizes physiological entrainment as a fundamental process that bridges bodily activity and cognitive, motor, and affective functioning.

In conclusion, the physiological entrainment phenomenon offers a functional understanding of mind–body interactions and can guide interventions that aim to improve cognitive, motor, and affective functioning. Exploring the therapeutic and rehabilitative potential of physiological entrainment remains crucial for designing targeted interventions that promote well-being. Future research should investigate the relevant relationship between contextual and cultural mechanisms and physiological entrainment. Moreover, future investigations should compare and evaluate the different methodological approaches to investigate the human entrainment phenomenon.

## Data Availability

Not applicable.
